# Soil bacterial and fungal community responses to nitrogen addition across soil depth and microhabitat in an arid shrubland

**DOI:** 10.3389/fmicb.2015.00891

**Published:** 2015-09-04

**Authors:** Rebecca C. Mueller, Jayne Belnap, Cheryl R. Kuske

**Affiliations:** ^1^Bioscience Division, Los Alamos National LaboratoryLos Alamos, NM, USA; ^2^Southwest Biological Science Center, United States Geological SurveyMoab, UT, USA

**Keywords:** dryland, shrubland, soil fungal community, soil bacterial community, global change, biological soil crusts, microhabitat, ribosomal RNA

## Abstract

Arid shrublands are stressful environments, typified by alkaline soils low in organic matter, with biologically-limiting extremes in water availability, temperature, and UV radiation. The widely-spaced plants and interspace biological soil crusts in these regions provide soil nutrients in a localized fashion, creating a mosaic pattern of plant- or crust-associated microhabitats with distinct nutrient composition. With sporadic and limited rainfall, nutrients are primarily retained in the shallow surface soil, patterning biological activity. We examined soil bacterial and fungal community responses to simulated nitrogen (N) deposition in an arid *Larrea tridentata*-*Ambrosia dumosa* field experiment in southern Nevada, USA, using high-throughput sequencing of ribosomal RNA genes. To examine potential interactions among the N application, microhabitat and soil depth, we sampled soils associated with shrub canopies and interspace biological crusts at two soil depths (0–0.5 or 0–10 cm) across the N-amendment gradient (0, 7, and 15 kg ha^−1^ yr^−1^). We hypothesized that localized compositional differences in soil microbiota would constrain the impacts of N addition to a microhabitat distribution that would reflect highly localized geochemical conditions and microbial community composition. The richness and community composition of both bacterial and fungal communities differed significantly by microhabitat and with soil depth in each microhabitat. Only bacterial communities exhibited significant responses to the N addition. Community composition correlated with microhabitat and depth differences in soil geochemical features. Given the distinct roles of soil bacteria and fungi in major nutrient cycles, the resilience of fungi and sensitivity of bacteria to N amendments suggests that increased N input predicted for many arid ecosystems could shift nutrient cycling toward pathways driven primarily by fungal communities.

## Introduction

Nitrogen (N) deposition due to human activities has increased dramatically and is predicted to rise with human population growth (Galloway et al., 2008). The effects of N additions on soil microbial communities have been well-studied in mesic forests in the eastern U.S., but the effects of adding N to dryland ecosystems are poorly understood. Arid lands have distinct characteristics compared to forests, with multiple limiting nutrients, including water, N, and P, and commonly have low inputs of carbon (Collins et al., 2008; Sterflinger et al., 2012), and it is unlikely that microbial responses to N can be accurately inferred from patterns documented in more mesic systems, particularly given documented interactions between soil properties and N responses. Historically, arid regions have received lower natural N inputs (~1–2 kg ha^−1^ yr^−1^; Phoenix et al., 2006), but it is not known if these lower levels have similar impacts comparable to higher inputs to more mesic regions. In addition, the native microbial communities in drylands are highly dissimilar from those found in mesic environments (Fierer et al., 2003; Dunbar et al., 2012). The effects of experimental climate change on soil microbial communities can depend upon the composition of the native community (Hawkes and Keitt, 2015), suggesting that variation among ecosystems could alter patterns of community structure and response to N deposition. Urban areas are sources of atmospheric N and are increasing in arid ecosystems (Galloway et al., 2004); thus, a future increase in N deposition is likely in these regions. Understanding soil microbial community responses to additional N input will provide insight on how we may expect the functions of dryland soils to change in response to future conditions, and how we may effectively manage nitrogen inputs into drylands to maintain productivity and resilience of microbial communities.

Drylands are typified by high heterogeneity in seasonal climate, the distribution of nutrients across the landscape, and physical microhabitats. Many hot dryland ecosystems experience strong seasonal shifts, with pronounced dry and rainy seasons and large fluctuations in temperature (Collins et al., 2008). These systems are also characterized by widely spaced vegetation, with biological soil crusts (biocrust) occupying the soil surface between the vascular plants (Belnap, 2003). Habitat heterogeneity has been shown to promote beta diversity across small geographic ranges for plants, insects, and microorganisms (Amarasekare, 2003), where differences in climatic factors are minimal. Previous studies of microbial community biomass and composition in dryland systems suggests these features are strongly correlated with differences in microhabitat (Garcia-Pichel et al., 2003; Schade and Hobbie, 2005; Bates and Garcia-Pichel, 2009; Steven et al., 2013, 2014), which can, in turn, shift function and subsequently alter microbial responses to changes in climate such as altered precipitation (Johnson et al., 2012; Delgado-Baquerizo et al., 2014). In addition, at a broad scale, microbial metabolic pathways have also been shown to differ between rhizosphere and biocrust soils (Steven et al., 2014).

Although most dryland soils do not have highly weathered soils with characteristic organic horizons, soil nutrients are highly stratified by depth (Pointing and Belnap, 2012). In addition, drylands are characterized by highly patchy distribution of vegetation (Collins et al., 2008), leading to high levels of heterogeneity at both vertical and horizontal scales. A full understanding of the microbial response and ecosystem effects of N addition in arid systems therefore requires examination of multiple microhabitats and soil depths. Strong vertical stratification of communities across shallow soil horizons has also been found for bacterial communities in dryland ecosystems (Kuske et al., 2002; Steven et al., 2013), with significant differences in communities between plant and crust-associated soils (Steven et al., 2013), but next generation sequencing technologies now provide the means for in-depth analysis of microbial community shifts. Using high throughput sequencing, we examined fungal and bacterial community response to N addition within two microhabitats: the plant root zone and interspaces soils covered by biocrusts. Soils were sampled at two depths in each microhabitat (0–0.5 cm surface veneer, and 0–10 cm soil).

Previous studies have documented patterns of bacterial or fungal composition in arid lands (e.g., Kuske et al., 2002; Bates et al., 2012; Steven et al., 2013, 2014), but have not examined the effects of N. In addition, few studies have utilized high-throughput sequencing methods to characterize microbial communities within drylands (Makhalanyane et al., 2015). Correlated shifts in bacterial communities, catabolic profiles, and metagenomic surveys have been shown in experimental N sites (Amarasekare, 2003; Fierer et al., 2012), but with no concurrent analysis of the fungal community. Although bacterial biomass generally exceeds that of fungal biomass in arid ecosystems (Bates and Garcia-Pichel, 2009), the majority of biogeochemical cycles in terrestrial ecosystems are driven by both bacteria and fungi (van der Heijden et al., 2008), so an understanding of the responses of both bacterial and fungal communities is needed to predict long-term ecosystem effects of N inputs. Consequently, we conducted compositional analysis of these two communities, combined with examination of nutrient transformations (Sinsabaugh et al., 2015), to provide insights into both the community- and ecosystem-level effects of N deposition on drylands.

## Materials and methods

The study site is located within the Lake Mead National Recreation Area in southern Nevada (36.009 N, 114.797 W). Two species of shrubs, *Larrea tridentate* and *Ambrosia dumosa* comprise the dominant vegetation, interspersed with patches of poorly developed biological soil crusts (biocrusts). Soils are alluvial, with high sand content (80%), low organic carbon (0.35%) and slightly basic pH (7.4 in bulk soils). Mean high and low annual temperatures are 23.8°C and 11.1°C, respectively, with mean annual precipitation of 19.8 cm based on data collected from a nearby weather station.

Within a 100 × 100 m plot, fifteen 2 × 2 m subplots were established, centered around similarly-sized *Ambrosia dumosa* shrubs. To examine the effects of N on plant and soil microbial communities, N was experimentally manipulated at two levels: 7 and 15 kg^−1^ ha^−1^ yr was added, along with an ambient control, hereafter referred to as N7, N15 and ambient. Nitrogen was applied in the form of ammonium nitrate in water every 2 months from March 2012 to May 2013, when soils were harvested.

**Figure 1:**
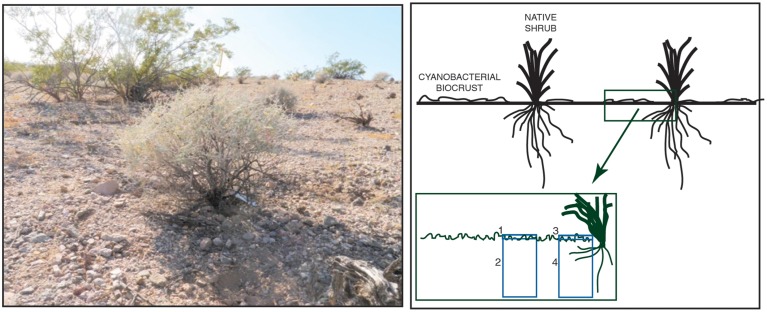
**Left**. Photo of *Ambrosia dumosa* shrub and surrounding interspace at the field site. **Right**. The sampling design used to examine bacterial and fungal communities in different locations in the soil.

To examine potential impacts of N amendment on bacterial and fungal community composition, we sampled soils from the two microhabitats (shrub and interspace) across two soil depths (surface and sub-surface) (Figure 1). Surface soils were sampled to a depth of 0.5 cm, and sub-surface soils were sampled to a depth of 10 cm. Five replicates per N treatment were collected from four sampling locations: shrub canopy surface at 0–0.5 cm depth (canopy biocrust), shrub canopy bulk soils at 0–10 cm depth (canopy bulk), interspace surface at 0–0.5 cm depth (interspace biocrust), and interspace bulk soil at 0–10 cm depth (interspace bulk). A total of 60 samples were collected. Soils were homogenized by hand and total nucleic acids were extracted from 0.5 g of soil using the MP Biomedical FastDNA for Soil kit (MP Biomedical, Santa Ana CA, USA) according to the manufacturer's instructions.

Bacterial and fungal communities were targeted for Illumina high-throughput sequencing using a two-stage PCR approach described previously (Mueller et al., 2014). The V3-V4 region of the bacterial 16S rRNA gene was amplified using the F515-R806 primer pair (Bates et al., 2010), and the D2 hyper-variable region of the fungal LSU rRNA gene was amplified using the newly designed LR22R primer (TAGCGMACAAGTASMGTGA) with the LR3 primer as the reverse (http://sites.biology.duke.edu/fungi/mycolab/primers.htm). Samples were barcoded to facilitate multiplexing using a combinatorial approach (Gloor et al., 2010), with a unique 6 bp barcode inserted into both the forward and reverse primer. Illumina-specific sequences were added in a second PCR. Amplicons were cleaned using the MoBio –htp UltraClean 96-well plate clean up kit according to the manufacturer's directions, but with a modification to reduce the amount of binding buffer from 5X to 3X sample volume to facilitate removal of longer primer-dimers. Individual samples were quantified using the Quant-It high-sensitivity double stranded DNA kit (Invitrogen, Carlsbad CA USA) using the BioTek Synergy H1 microplate reader and combined at equimolar concentrations. The multiplexed library was cleaned using the MoBio UltraClean PCR clean up kit and eluted in 50 μl EB buffer. Dissimilar values from fluorometry and Illumina-specific qPCR indicated that the second PCR step did not add the Illumina-specific primers to all samples, and a final PCR, with conditions identical to the second PCR, was performed on the library, for a total of three cycles. Library length and concentration was verified using a bioanalyzer and qPCR, respectively. Amplicons were sequenced using paired end 250 bp reads on the Illumina MiSeq platform at Los Alamos National Laboratory. To offset the low diversity of nucleotides in the initial sequence common in amplicon sequences, genomic DNA from multiple bacterial isolates was added at approximately 30%.

Bacterial and fungal community sequence datasets were de-multiplexed individually using QIIME (Caporaso et al., 2010b), with quality filtering to remove any sequence with a mismatch to the barcode or primer sequence. Downstream sequence processing was conducted using UPARSE (Edgar, 2013). Sequences within an expected error greater than 0.5 and singleton sequences were removed. Putative chimeras were identified *de novo* using UCHIME (Edgar et al., 2011) and removed, and OTUs were delineated at 97% sequence similarity. The OTU table was exported, and all statistical analyses were conducted using the R statistical platform (r-sourceforge.org). Representative fungal and bacterial sequences were selected and classified against their respective Ribosomal Database Project (RDP) databases using the online tool. Any sequence identified as non-Fungal or non-Bacterial was removed from subsequent analyses. Sequences were deposited to the MG-RAST server (Meyer et al., 2008) under IDs 4635375.3 (Bacteria) and 4635376.3 (Fungi).

To construct phylogenetic trees, we utilized reference phylogenies constructed using partial to full-length ribosomal sequences. For the bacteria, we used the tree from Kembel et al. (2012), and for fungi we used a tree constructed from fungal sequences generated by the Assembling the Fungal Tree of Life Project (Celio et al., 2007). Representative OTU sequences were aligned to the reference taxa using PyNAST (Caporaso et al., 2010a) and placed on their respective reference phylogenies using pplacer (Matsen et al., 2010).

Statistical analyses were conducted using the R libraries picante (Kembel et al., 2010), vegan (Oksanen et al., 2013), and phyloseq (McMurdie and Holmes, 2013). Analyses were conducted by randomly selecting 5200 sequences for bacteria and 4500 sequences for fungi, with 99 trials for each analysis to limit bias resulting from random sampling of communities. Three bacterial and two fungal samples had insufficient sequencing depth and were not included in the analyses. Richness measures, including OTU richness and phylogenetic diversity (PD) (Faith, 1992) were compared using Three-Way ANOVA with N treatment, microhabitat, and soil depth as fixed factors. Community composition based on OTU similarity (Bray-Curtis dissimilarity metric) and phylogenetic similarity using the weighted UniFrac distance (Lozupone and Knight, 2005) were compared using PERMANOVA with all three factors included in the model (Anderson, 2001). In addition, to examine responses at broader taxonomic scales, we grouped fungal and bacterial OTUs at class and phylum levels, respectively, and tested for significant differences in relative abundance across N treatment, soil depth, and microhabitat using Three-Way ANOVA.

## Results

**Figure 2:**
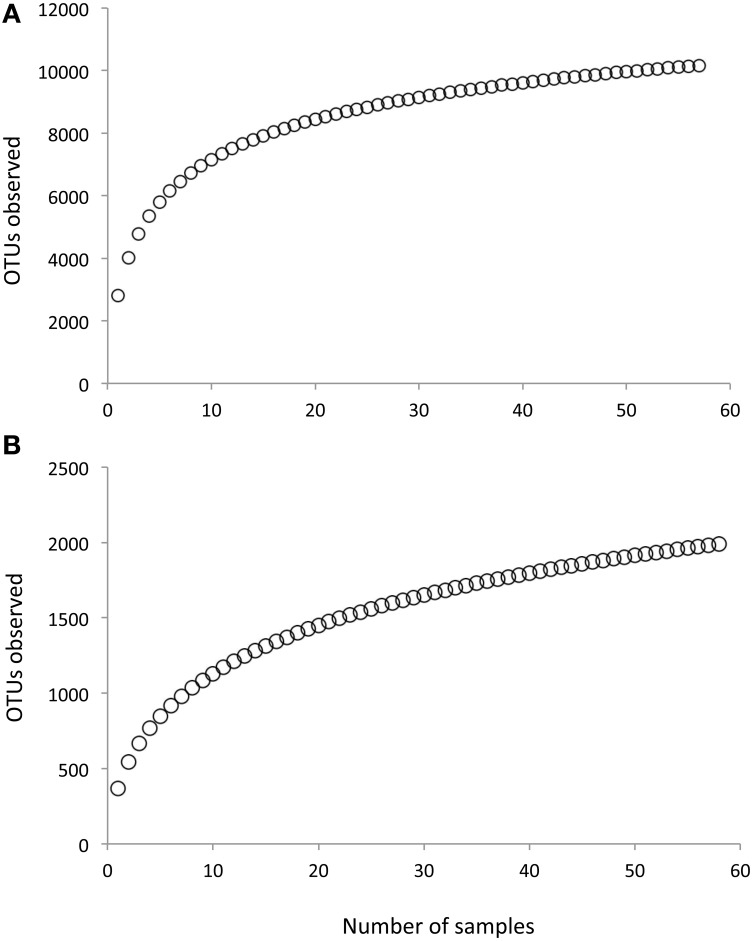
**Rarefaction curves of (A) Fungal communities and (B) Bacterial communities**.

Following removal of low-quality sequences, a total of 1,340,717 fungal LSU sequences and 1,466,336 bacterial 16S sequences were included in the analysis. Across all samples, we observed 1990 fungal OTUs and 10167 bacterial OTUs. Bootstrap estimates of gamma diversity indicated that both communities were slightly under-sampled; the bootstrap estimate was 2226 (±45 SE) for fungi and 10884 (±160 SE) for bacteria (Figure 2).

### Effects of microhabitat and soil depth

**Figure 3:**
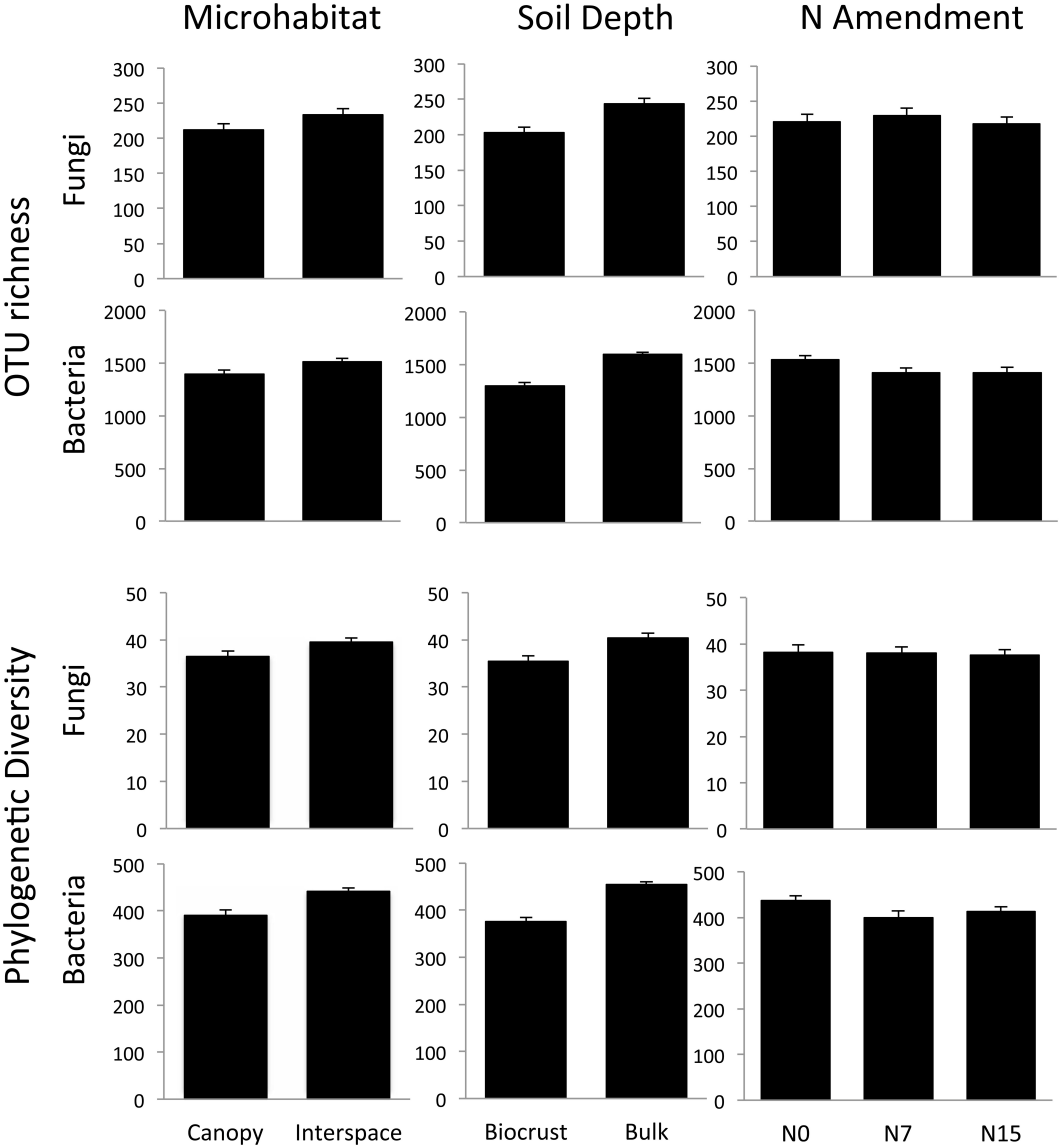
**OTU richness and phylogenetic diversity (Faith's PD) in the N-amended plots across different soil depths and microhabitats**. Both communities showed significant differences across soil depths and microhabitats, but only bacteria showed significant shifts in response to N. While results are illustrated here by individual treatment, the results were analyzed using a Three-Way ANOVA framework. See Results text for statistical support.

Microhabitat and soil depth each had strong effects on fungal and bacterial community richness and PD. For the fungal community, soil depth was the strongest driver of diversity differences for both OTU richness (*F* = 128, *p* < 0.001) and PD (*F* = 11.7, *p* = 0.001). Surprisingly, richness and PD were higher in the bulk soil depth (Figure 3). OTU richness did not vary significantly between the shrub canopy and the interspace (*F* = 3.34, *p* = 0.07), although there was a trend for higher fungal richness in association with interspace compared to shrub canopy soils (Figure 3). However, PD of fungal communities associated with the interspaces was significantly higher than those found in the shrub canopy (*F* = 4.42, *p* = 0.04).

The bacterial community also showed strong differences in relation to soil depth. As with the fungal community, OTU richness (*F* = 140, *p* < 0.001) and PD (*F* = 137, *p* < 0.001) were significantly higher in the deeper bulk soils (Figure 3). Bacterial richness also differed significantly between the two microhabitats, with higher richness (*F* = 0.24, *p* < 0.001) and PD (*F* = 58, *p* < 0.001) observed in the interspaces than in the shrub canopy when analyzed across both soil depths.

**Figure 4:**
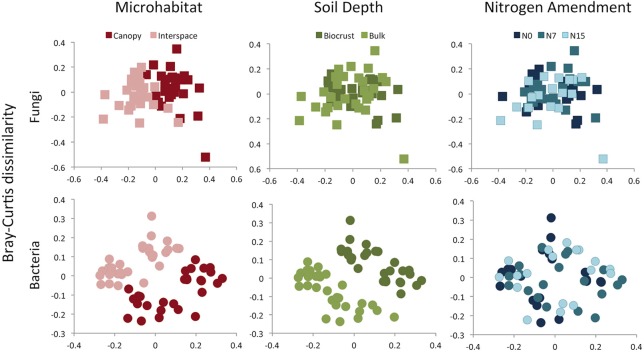
**Bray-Curtis dissimilarity among fungal or bacterial communities, displayed as 2D MDS plots**. Plots are color coded to show microhabitat (red), soil depth (green), or N amendment (blue) differences. While results are presented for individual treatments, results were analyzed using a three-way framework with PERMANOVA. See Results text for statistical support.

**Figure 5:**
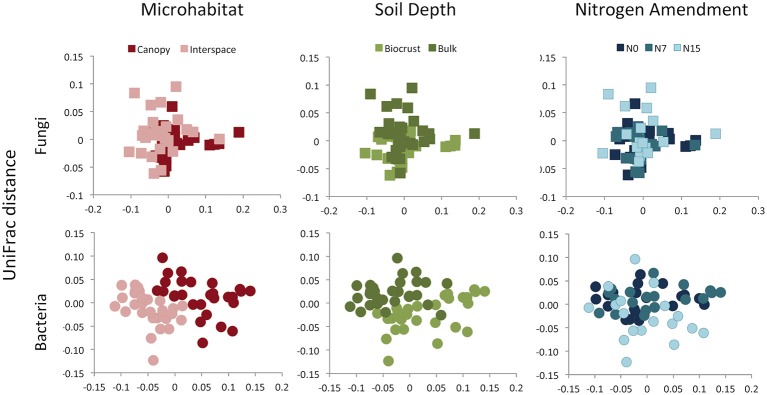
**Community composition (UniFrac distance) of fungal or bacterial communities, displayed as 2D MDS plots**. Plots are color coded to show microhabitat (red), soil depth (green), or N amendment (blue) differences. See Results text for statistical support.

Although for fungal and bacterial communities, the strongest richness differences were found between the two soil depths, the largest driver of variation in community composition was microhabitat (Figures 4, 5). This pattern was observed using both OTU-based measures (Fungi: *F* = 8.89, R2 = 0.13, *p* = 0.001, Bacteria: *F* = 6.10, R2 = 0.10, *p* = 0.001) and phylogenetic-based measures (Fungi: *F* = 11.6, R2 = 0.15, *p* = 0.001, Bacteria: *F* = 23.9, R2 = 0.23, *p* = 0.001). Both communities were also delineated by soil depth, but this factor explained a lower proportion of community variation for both OTU-based (Fungi: *F* = 2.50, R2 = 0.04, *p* = 0.001, Bacteria: *F* = 2.62, R2 = 0.04, *p* = 0.03) (Figure 4) and phylogenetic measures (Fungi: *F* = 9.49, R2 = 0.12, *p* = 0.001, Bacteria: *F* = 15.7, R2 = 0.15, *p* = 0.001) of community similarity (Figure 5).

### Nitrogen effects on microbial communities

We found no evidence for fungal community responses to N addition. We observed no shifts in OTU richness in response to N addition (*F* = 0.48, *p* = 0.62) and no change in Faith's PD (*F* = 0.11, *p* = 0.90) (Figures 4, 5). Neither OTU-based community composition (*F* = 1.17, R2 = 0.04, *p* = 0.18), nor phylogenetic composition (*F* = 1.18, R2 = 0.04, *p* = 0.27) shifted in response to experimental N additions (Figures 4, 5).

In contrast to responses in the fungal community, we found consistent negative responses to N in the bacterial community diversity indices (Figure 3). Both richness (*F* = 9.85, *p* < 0.001) and PD (*F* = 11.7, *p* < 0.001) declined in N-amended plots. Tukey's Honestly Significant test indicated that for both measures, intermediate and high levels of N (N7 and N15) were not significantly different (*p* > 0.2 for each). Nitrogen addition was associated with community shifts using both OTU-based (*F* = 1.34, R2 = 0.04, *p* = 0.02) and phylogenetic measures (*F* = 3.42, R2 = 0.07, *p* = 0.001; Figure 4). Using the UniFrac distance measure, we also found a significant N X soil horizon depth interaction (*F* = 1.87, R2 = 0.04, *p* = 0.04), where community shifts were more pronounced at lower soil depths.

### Broad taxonomic responses

**Figure 6:**
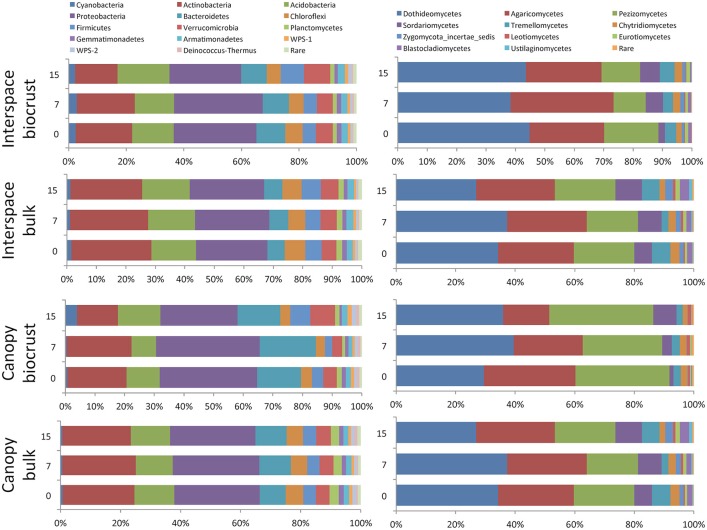
**Relative abundance of different bacterial phyla (left) and fungal classes (right)**. Only groups with a minimum relative abundance of 1% are shown.

The phylum Ascomycota was the dominant fungal phylum within this shrubland. At finer taxonomic scales, the most abundant fungal classes included the Dothideomycetes, Pezizomycetes, and the Basidiomycete class Agaricomycetes. The bacterial community was dominated by the Proteobacteria, followed by the Actinobacteria and Acidobacteria (Figure 6).

When bacterial and fungal communities were examined by grouping OTUs into bacterial phyla or fungal classes, respectively, we found that only a single fungal class, the Sordariomycetes, showed a response to N addition, with a nearly linear increase in relative abundance in N amended plots (*F* = 4.34, *p* = 0.02). Three classes, the Leotiomycetes, Sordariomycetes, and Eurotiomycetes, were all significantly more abundant at deeper soil depths (for each, *p* < 0.03). No significant effect of microhabitat on the abundance of any fungal class was observed.

In contrast, numerous bacterial phyla showed significant shifts in relative abundance in response to N additions (Table 1). The two most abundant groups, the Actinobacteria and Proteobacteria, showed negative responses at the highest level of N input. Responses for the classes within the Proteobacteria were consistent, with declines observed for alpha, beta, delta, gamma, and epsilon classes. Four other bacterial phyla, the Acidobacteria, Chloroflexi, Firmicutes, and Verrucomicrobia, showed significant positive responses to N amendment. In addition, a number of bacterial phyla showed significant differences across soil depth and microhabitat type. The relative abundance of most phyla was higher at lower soil depths, with the exception of Proteobacteria and Verrucomicrobia, which exhibited higher relative abundance in the surface soils. Microhabitat also affected a large number of bacterial phyla. For example, the relative abundance of Acidobacteria was higher in the interspace soils, while the proportion of Bacteroidetes was higher in shrub-associated soils (Table 1).

**Table 1:** *****F***-values from a Three-Way ANOVA comparing the abundance of different bacterial phyla in response to nitrogen(N) addition, soil depth, and microhabitat**.

	**N**	**N response**	**Soil depth**	**Depth response**	**Microhabitat**	**Micro- response**
Acidobacteria^§^	13.7^***^	↑	6.0 ^**^	L	65.5^***^	I
Actinobacteria	10.1^***^	↓	57.2^***^	L	0.90	
Armatimonadetes^±^	1.14		0.11		46.0^***^	I
Bacteroidetes^±^	2.91		73.9^***^	U	92.3^***^	C
Chloroflexi	4.59^*^	↓	55.6^***^	L	45.8^***^	I
Firmicutes^§^	19.0^***^	↑	0.01		23.2^***^	I
Planctomycetes	1.52		58.2^***^	L	15.4^***^	C
Proteobacteria^§^	9.62^***^	↓	18.7^***^	U	29.4^***^	C
Verrucomicrobia^§^	12.1^***^	↑	6.80^*^	U	10.2 ^**^	I

Significance levels are represented as

* p < 0.05,

** p < 0.01,

*** *p < 0.001. Nitrogen response indicates the overall trend of N response (positive or negative). Depth and microhabitat response indicates the factor where the highest abundance was observed, where U indicates upper biocrust soil layer, L indicates lower bulk soil layer, I indicates interspace soils, C indicates shrub canopy soils*.

§ *Indicates a significant interaction between N and soil depth*.

± *Indicates a significant interaction between soil depth and microhabitat*.

*The arrows correspond to positive or negative responses to nitrogen*.

## Discussion

Our study objective was to determine the cumulative impacts of N deposition, delivered every 2 months for 14 months, on the microbial communities within an arid shrubland ecosystem. Because of its mosaic nature, we partitioned the landscape into compartments and separately assessed the N amendment impacts on those compartments. This study represents the first high-depth-of-coverage, taxonomic survey of both the fungal and bacterial communities in four shrubland microhabitats—shrub canopy and interspace soils, each stratified at two shallow depths. We also examined the sensitivity of those fungal and bacterial communities to short-term (1 yr), relatively low concentrations of N amendment.

### Fungal and bacterial community differences with soil depth

In a companion report to this study, bacterial and fungal biomass, measured by qPCR, was significantly higher in the 0–0.5 cm biocrusts than in 0–10 cm depth bulk soil (>100% and >200% higher, respectively; Sinsabaugh et al., 2015). This is a typical pattern for arid shrublands and grasslands, where, by multiple measures, microbial biomass has been found to be concentrated in the upper few cm of soil (Dunbar et al., 2012; Pointing and Belnap, 2012; Steven et al., 2013). Biocrust biomass may differ substantially with soil type (Steven et al., 2012) and with biocrust developmental stage (Garcia-Pichel et al., 2003; Yeager et al., 2004), particularly in Cyanobacterial biocrusts where the proportion and density of the dominant cyanobacteria in the biocrust controls the overall biomass. Our study partitioned soil depth into intervals at the cm scale; however, biomass of biocrust soils is stratified even at the mm interval (Garcia-Pichel et al., 2003).

Although biomass (qPCR) was higher in the upper soil depths, the OTU-based richness for both fungal and bacterial communities was higher in the 0–10 cm depth interval compared to the 0–0.5 cm biocrust. We found no significant interaction between depth and microhabitat, indicating that this pattern was consistent for biocrusts and shrub canopy locations (Figure 2). This pattern has been found in prior studies of arid land biocrusts, where the dominance of a few cyanobacterial sequence types (often 40–60% of the total sequences) is associated with lower richness estimates (Johnson et al., 2012; Steven et al., 2013) relative to soils below the biocrust. A contrasting feature of this shrubland is that the relative abundance of Cyanobacteria within the biocrusts is very low (~3%; Figure 6), indicating that the biocrusts here are poorly developed. As a result, it is possible that higher bacterial and fungal richness at lower soil depths is due to UV radiation stress coupled with drier soil conditions and wider temperature swings experienced by other soil organisms in the upper soil layers. For the fungal community, we also found evidence for selection of UV tolerant taxa in the upper soil depths relative to the deeper soils, with higher abundance of the orders Pleosporales and Dothidiales, which contain numerous species of melanized fungi (Sterflinger et al., 2012). Evaporation is likely greater in upper soil layers, and previous studies have shown that bacterial richness is strongly correlated with soil moisture in hyperarid sites, with decreasing diversity as moisture becomes increasingly limiting (Crits-Christoph et al., 2013).

For both the bacterial and fungal communities, broad taxonomic groups showed different patterns across soil depth. With the exception of Cyanobacteria noted above, the phylum level distribution of bacteria by depth is parallel to that reported by Steven et al. (2014), who showed that for three different soil types covered by well-developed biocrusts, the relative abundance of Acidobacteria, Actinobacteria, and Chloroflexi were higher below the surface biocrust than within the biocrust. Class level comparisons within the fungi showed higher abundance of the Leotiomycetes, Sordariomycetes, and Eurotiomycetes in the bulk soil when compared to the biocrust, with no significant shift in other classes (Figure 6). For example, the relative abundance of the Eurotiomycetes was double in the lower soil depths compared to the surface layers (1.25% and 0.61%, respectively). It is also notable that we observed higher abundance of the Agaricomycetes at this site compared to previous studies in drylands (Bates and Garcia-Pichel, 2009; Steven et al., 2014), although this class did not show significant responses to any of the three factors analyzed.

### Fungal and bacterial community differences between shrub canopies and interspaces

Based on qPCR assays conducted at this site, fungal and bacterial biomass was higher in the shrub canopy soils than in the interspace soils (Sinsabaugh et al., 2015). In the current study, we found that the richness of both fungi and bacteria was higher in interspace soils compared to shrub-associated soils (Figure 3). This differs from the findings of Steven et al. (2014) conducted at a nearby site in the Mojave Desert in Nevada, in which fungal and bacterial richness was higher in shrub root zones than in the interspaces. This could be due to differences in biocrusts between this and the current study; the biological crusts examined in Steven et al. (2014) were well-developed and dominated by a high biomass of Cyanobacteria, whereas the relative abundance of cyanobacterial sequences in the biocrusts in the current study was relatively low, with greater dominance by Proteobacteria and Actinobacteria, thus more closely resembling the communities found at 0–10 cm soils in the Steven et al. (2014) study. Because the bacterial community is not dominated by a single phylum (Cyanobacteria) within the biocrusts at our site, it is possible that the different responses observed are linked with variability in competitive interactions between fungi and bacteria (Boer et al., 2005) within well-developed biocrusts compared to bulk soils, either directly via resource competition, or through the different types of carbon inputs in well-developed crusts (e.g., derived from Cyanobacteria).

In addition to contributing to differences in richness, microhabitat was the strongest driver of shifts in community composition for both fungi and bacteria (Figures 4, 5). The role of plants in altering microbial communities has been documented in numerous dryland systems, where communities associated with the rhizosphere were distinct from those found in un-vegetated soils (Andrew et al., 2012; Ramond et al., 2014; Steven et al., 2014). Plants have often been considered to be “islands of fertility” in arid ecosystems, and while biomass is often higher in the rhizosphere (e.g., Pointing and Belnap, 2012), Ben-David et al. (2011) found no significant differences in bacterial biomass between shrubs and interspace soils in the arid portion of the Negev desert. In the current study, we found that bulk soils can harbor diverse bacterial and fungal communities, but plants appear to select for a specific group of microbial taxa.

### Soil community responses to N amendment

Fungal and bacterial communities exhibited differential responses to N amendment. Fungal community biomass as measured by rRNA gene qPCR (Sinsabaugh et al., 2015) and richness did not change significantly with N amendment (Figure 3). Compositionally, members of the fourth most abundant fungal class, the Sordariomycetes, showed significant positive responses to N addition (Figure 6). Lauber et al. (2008) showed that members of this class residing in forest and pasture soils were higher in soils with higher P content and with higher C:N ratio. In our study site, N amendment significantly increased available N and P by 30 and 25% respectively, over the ambient condition (Sinsabaugh et al., 2015), and it is possible that the observed increase in Sordariomycete is a result of N or P no longer being limiting for these fungi.

In contrast to the limited responses exhibited by the fungal community, the bacterial community responded with changes in richness and composition. Bacterial richness declined with increasing levels of N (Figure 3), and the relative abundance of multiple bacterial phyla shifted with N amendment (Table 1). However, the bacterial composition response patterns to N amendment we observed are not consistent with previously hypothesized ecological strategies of soil bacteria (Fierer et al., 2007) or taxonomic response patterns for these phyla under N amendment in two mesic grasslands (Fierer et al., 2012; Ramirez et al., 2012). Where culturable, the Acidobacteria, and Verrucomicrobia have been typified by small cells, slow growth, and ability to use a wide variety of complex carbon substrates; thus, they could be considered oligotrophs (Fierer et al., 2007). On the other hand, the Firmicutes may exhibit rapid growth followed by abundant spore formation and are generally considered copiotrophs. All three phyla contain species that can persist for extended periods of time in the soil in dormant states, becoming active under favorable conditions (Lennon and Jones, 2011). We found significant increases in the relative abundance of the Acidobacteria, Firmicutes, and Verrucomicrobia phyla despite their different ecological strategies. In contrast, we found that the relative abundance of Actinobacteria, Chloroflexi, and Proteobacteria decreased in soils due to increased soil N. There are few commonalities among and within these three phyla with regard to growth rates or known carbon use lifestyles (oligotrophs vs. copiotrophs) in the soil, indicating that in this arid ecosystem, responses cannot be readily predicted based on *a priori* estimates of ecological strategies. This could be due to differences among ecosystems (e.g., mesic vs. arid) or as a result of greater variability in ecological strategy within bacterial phyla than has been previously observed.

One of the most striking patterns observed was that N amendment resulted in a decrease in the relative abundance of two dominant phyla, the Proteobacteria and Actinobacteria. Previous studies on plant communities have found that rare taxa tend to be susceptible to N deposition (Shaw et al., 2002; Clark and Tilman, 2008). Here, we show the opposite response pattern in bacterial communities. The decreased abundance of the Proteobacteria may be of particular importance in drylands, as members of the phylum have been shown to promote plant growth and facilitate horizontal transfer to genes involved in photosynthesis (Makhalanyane et al., 2015). The driver of this unexpected response could be complex interactions between resource availability and microbial responses, or could be due to phenotypic plasticity under variable environmental conditions, such that closely related organisms in different environments exhibit different traits. Alternatively, N deposition could shift competitive interactions within the bacterial community, leading to taxonomic losses in abundant groups, such as the Proteobacteria. Different responses to N based on broad functional groups have been observed across previous studies. A recent meta-analysis found that N deposition increased autotrophic respiration by 22%, with a concurrent 13% decrease in heterotrophic respiration (Zhou et al., 2014).

The physiological underpinnings of the different, yet dramatic response of bacteria among experimental N-amendment studies remain unknown, and are difficult to infer or generalize at the phylum level. Possibilities include different within-phylum species composition among the different soils tested, different responses to N amendment in the alkaline soil of the arid shrubland compared to the acidic soils in more mesic settings, and *differences* due to inherent organic matter or elemental concentrations in the soils. Indeed, Liu and Greaver (2009) noted in a review article that soil microbial respiration increased in grassland biomes but tended to decrease in forest biomes with N additions. Similarly, Janssens et al. (2010), found that although soil respiration decreased an average of 15% across many forests with an N amendment, the response range was huge, ranging from −57% to +63%. Clearly, studies incorporating both community and process analyses are required to determine key responsive populations and soil process responses to altered N deposition.

### Implications for ecosystem processes

Based on a companion study conducted at the same site, aspects of the N cycle (N mineralization, nitrification, and ammonification) were all higher in the biocrust compared to the deeper bulk soils, and only the upper biocrust layer showed a significant response to N addition (Sinsabaugh et al., 2015). Diversity has been linked with increased nutrient cycling rates (Philippot et al., 2013), but particular functional groups, such as denitrifying bacteria, are often studied in isolation. In natural communities with multiple co-existing functional groups, nutrient cycling rates are likely due not only to species richness, but also to interacting factors, such as the strength of competitive interactions or species traits such as community niche (Salles et al., 2009). For example, *Fusarium*, a fungal genus in the class Sordariomycetes, is known to function in denitrification. The Sordariomycetes was the only fungal class that responded to N addition; however, we also found higher richness in the lower soil depths with added N. In addition, fungal isolates capable of denitrification are widely distributed across the fungal phylogeny in classes such as the Eurotiomycetes and Leotiomycetes (Jasrotia et al., 2014).

Despite limited direct links between community measures and nutrient cycling, our findings have potential implications for pathways of N cycling within this system. The lack of response in the fungal community with strong shifts in the bacterial community suggests that increased N availability will likely shift toward fungal-dominated pathways in N-amended soils. Fungi and bacteria have different functional roles and patterns of resource utilization related to decomposition (Schneider et al., 2012), and shifts in the relative abundance of these two groups will likely alter biogeochemical cycles (Litchman et al., 2015). Whether community shifts observed will alter decomposition in this system is less clear. Nitrogen addition has been shown to increase decomposition rates, but this is not mediated by the initial phylogenetic relatedness of the fungal community (Amend et al., 2015). However, the realized composition of the fungal community was not measured in this study, and so the links between fungal communities and decomposition could not be compared directly. Fungi can operate at much lower soil water potentials (Chen et al., 2015), and so the functional roles of fungi in dryland ecosystems differ from the well-studied roles of fungi within mesic areas, particularly in terms of carbon utilization (Makhalanyane et al., 2015), denitrification (Laughlin and Stevens, 2002; Crenshaw et al., 2007; Chen et al., 2015), and potential lateral nutrient transfer among biocrusts and plants (Green et al., 2008). The findings of the current study illustrate that nitrogen effects on microbial communities are complex, and responses observed in one ecosystem may not be apparent in others. The impacts of nitrogen in terrestrial ecosystems are likely to occur across large portions of the globe, suggesting that increased efforts to quantify effects on microbial communities in less understood ecosystems will provide important insights into how nitrogen inputs will alter ecosystem functions at landscape scales.

## Funding

Funding for the field research was provided by the National Park Service (Lake Mead). JB also acknowledges the support of USGS Ecosystems and Climate Change and Land Use programs. CK and RM and the sequencing activities were supported by a Science Focus Area grant to Los Alamos National Laboratory by the US Department of Energy, Office of Science, Biological and Environmental Research Division, and a Director's Postdoctoral Fellowship to RM. Any use of trade names is for descriptive purposes only and does not imply endorsement by the U.S. Government.

## Conflict of interest statement

The authors declare that the research was conducted in the absence of any commercial or financial relationships that could be construed as a potential conflict of interest.
